# Genetic associations of risk behaviours and educational achievement

**DOI:** 10.1038/s42003-024-06091-y

**Published:** 2024-04-10

**Authors:** Michelle Arellano Spano, Tim T. Morris, Neil M. Davies, Amanda Hughes

**Affiliations:** 1 Medical Research Council Integrative Epidemiology Unit at the University of Bristol, Bristol, BS8 2BN United Kingdom; 2 Population Health Sciences, Bristol Medical School, University of Bristol, Barley House, Oakfield Grove, Bristol, BS8 2BN United Kingdom; 3 Centre for Longitudinal Studies, Social Research Institute, University College London, London, United Kingdom; 4 Division of Psychiatry, University College London, Maple House, 149 Tottenham Court Rd, London, W1T 7NF United Kingdom; 5 Department of Statistical Sciences, University College London, London, WC1E 6BT United Kingdom; 6 K.G. Jebsen Center for Genetic Epidemiology, Department of Public Health and Nursing, Norwegian University of Science and Technology, 7491 Trondheim, Norway

**Keywords:** Genetics research, Epidemiology

## Abstract

Risk behaviours are common in adolescent and persist into adulthood, people who engage in more risk behaviours are more likely to have lower educational attainment. We applied genetic causal inference methods to explore the causal relationship between adolescent risk behaviours and educational achievement. Risk behaviours were phenotypically associated with educational achievement at age 16 after adjusting for confounders (−0.11, 95%CI: −0.11, −0.09). Genomic-based restricted maximum likelihood (GREML) results indicated that both traits were heritable and have a shared genetic architecture (Risk ${{{{{{\boldsymbol{h}}}}}}}^{{{{{{\boldsymbol{2}}}}}}}$ = 0.18, 95% CI: −0.11,0.47; education ${{{{{{\boldsymbol{h}}}}}}}^{{{{{{\boldsymbol{2}}}}}}}$ = 0.60, 95%CI: 0.50,0.70). Consistent with the phenotypic results, genetic variation associated with risk behaviour was negatively associated with education (${{{{{{\boldsymbol{r}}}}}}}_{{{{{{\boldsymbol{g}}}}}}}$ = −0.51, 95%CI: −1.04,0.02). Lastly, the bidirectional MR results indicate that educational achievement or a closely related trait is likely to affect risk behaviours PGI (*β*=−1.04, 95% CI: −1.41, −0.67), but we found little evidence that the genetic variation associated with risk behaviours affected educational achievement (*β*=0.00, 95% CI: −0.24,0.24). The results suggest engagement in risk behaviour may be partly driven by educational achievement or a closely related trait.

## Abstract (web summary)

A genetic study that explores the relationship between risk behaviours engagement in adolescents and educational achievement. This study found that risk behaviours were phenotypically associated with educational achievement at age 16, even after adjusting for confounders.

## Introduction

Risk behaviours like alcohol use, smoking and physical inactivity are often first engaged in adolescence and persist into adulthood^1^ Adolescence is a crucial formative period for an individual’s future well-being; the choices made during this period can have important repercussions later in life^2^ For example, greater engagement in risk behaviours at a young age is associated with increased risk of injury, substance dependence and lower educational attainment^3,4^ Evidence suggests that for each additional risk behaviour adolescents partake in, the odds of attaining five A*-C grades (a common marker of enrolment in further education and entry to skilled employment) at age 16 are 23% lower. If causal, risk behaviours in adolescence could, therefore, be a key target for interventions aiming to improve socioeconomic and health outcomes.

Risk behaviours tend to cluster and co-occur within individuals. This clustering can occur because of various reasons. First, engagement in one behaviour can lead to engagement in other risk behaviours, in a process known as co-occurrence^5^ For example, alcohol use can increase the risk of risky sexual behaviours via inhibition mechanisms affecting an individual’s decision-making processes^6^ The aforementioned effect, where one behaviour causes the other, was also demonstrated by ref. 7, who observed that early substance use was associated with an increased risk of engaging in premature sexual activity in adolescent girls. Similarly, features of an adolescent’s social and psychological environment, such as peers’ behaviour, can simultaneously influence engagement in multiple risk behaviours (environmental confounding)^8^ One source of environmental confounding are indirect genetic effects ('dynastic' effects or 'genetic nurture'), which occurs when relatives’ heritable traits affect children’s outcomes through environmental pathways. This bias is particularly evident in genetic studies of intergenerational transmission of education. Genetically influenced traits associated with educational achievement in the parents’ generation may lead to environments which promote educational achievement in children^9^. Such passive gene-environment correlation can impact the children’s educational achievement via environmental pathways, alongside any effects due to direct genetic inheritance, and inducing confounding through a correlation between genotypes and phenotypes.

The literature has focused on the effect of risk behaviours on various behavioural and social outcomes. These report associations between risk behaviours in adolescence and socioeconomic position later in life^10^, adult aggression^11^ and continuity of substance misuse^12^ However, it is unclear whether risk behaviours causally affect educational achievement or if features of the environment (confounding) influence both^13^ or educational achievement influencing risk behaviours (reverse causation) ^14,15^. Genetically informed studies can help overcome these sources of bias and improve our understanding of the causal relationships between education and risk behaviours in adolescents.

This study assessed the bidirectional causal relationships between adolescent risk behaviour and educational achievement. We applied genetic methods to study the genetic architecture of risk behaviours and educational achievement in an English cohort. We implemented a bidirectional Mendelian randomisation (MR) to investigate the causal direction of associations between these traits since a causal effect between education and risk is plausible in either direction. To minimise confounding and reverse causation, we use a polygenic risk indices (PGI) to capture risk and education liability.

## Results

### Sample description

We began with the original ALSPAC sample of 15,645 pregnancies, which was then restricted to those with genetic data and National Pupil Database linkage available. We subsequently excluded participants with consent withdrawals, participants not alive at 1 year, and those with no recorded sex and no socioeconomic information (maternal education and housing tenure). This process yielded a final analytical sample of 7695 participants, of whom 51% were male and 49% female. The phenotypic and MR analyses were carried out using imputed data on these 7695 participants. Of these, 1583 participants had complete information on all risk behaviours and covariates. This complete case sample was used for GREML analyses. Table 1 in the supplementary material shows the differences across the risk behaviour index and covariates between this complete case sample (*N* = 1583) and the remainder of the original ALSPAC sample (*N* = 14,062).

**Table 1:** Associations of capped GCSE score with the MRB index, based on imputed data (*N* = 7695)

Capped GCSE score	Model 1^a^	Model 2^b^	Model 3^c^	Model 4^d^
95% confidence intervals in brackets				
MRB Index	−0.14 [−0.17, −0.12]	−0.12 [−0.14, −0.10]	−0.12 [−0.13, −0.10]	−0.11[−0.13, −0.09]

^a^Model 1 is unadjusted for any covariates.

^b^Model 2 is adjusted for parental socioeconomic position, maternal education (ref:<O level) and sex (ref: male).

^c^Model 3 is adjusted for parental socioeconomic position, maternal education (ref:<O level), sex (ref: male) and housing tenure (ref: owned).

^d^Model 4 is adjusted for parental socioeconomic position, maternal education (ref:<O level), sex (ref: male), housing tenure (ref: owned) and cognitive ability.

### Phenotypic associations of risk behaviours and educational achievement

Table 1 reports results from models where we regress the capped GCSE score on the MRB Index using imputed data. The first column shows the regression results of the capped GCSE score on the MRB Index unadjusted for any covariate. A standard deviation increase in the MRB Index was phenotypically associated with a 0.14 (95% CI: [0.12, 0.17]) standard deviation decrease in capped GCSE score. After adjusting for sex, parental socioeconomic position and maternal education, a standard deviation increase in the MRB Index corresponds to a 0.12 (95% CI: [0.10, 0.14]) standard deviation decrease in capped GCSE score. This finding suggests that engagement in risk behaviour is associated with lower capped GCSE scores net of covariates. Likewise, results for the fully adjusted binary outcome model suggested the odds of obtaining five or more A*-C GCSEs were 19% (95% CI: [16, 23%]) lower per standard deviation increase (see supplementary, Table 6).

### Genotypic associations of risk behaviours and educational achievement

The univariate GREML models show associations between the phenotypes of interest and the genotypic data (Table 2). We observed SNP heritability in the educational achievement of 0.60 (95%CI: [0.50, 0.70]) for the capped GCSE score (continuous measure). The estimated heritability of the MRB Index was lower at 0.18 (95%CI: [−0.11, 0.47]), and the confidence interval crossed the null. These results suggest that considerable variation in the educational achievement measures can be explained by common genetic variation and provide weaker evidence that some variation in the risk behaviour index can be explained by common genetic variation.

**Table 2:** GCTA estimates. ${h}^{2}$: univariate heritability, ${r}_{g}$: genetic correlation

Univariate estimates	*n*	${h}^{2}$^a^	SE	95% CI	
MRB index	2171	0.18	0.15	−0.11	0.47
Capped GCSE score ^b^	6646	0.60	0.05	0.50	0.70
Bivariate estimates	*n*	${r}_{g}\,$^c^	SE	95% CI	
Capped GCSE score: MRB index	4409	−0.51	0.27	−1.04	0.02

^a^${h}^{2}$ shows the univariate heritability of each item.

^b^Capped GCSE is a continuous measure of educational achievement.

^c^$${r}_{g}$$ genetic correlation.

The bivariate GREML models show a strong negative genetic correlation between the MRB index and educational achievement of −0.51 (95%CI: [−1.04, 0.02]) for the capped GCSE score. This result suggests considerable genetic overlap between these traits and that genetic variation associated with risk behaviours is also associated with lower educational achievement.

### Bidirectional Mendelian randomisation

**Fig. 1:**
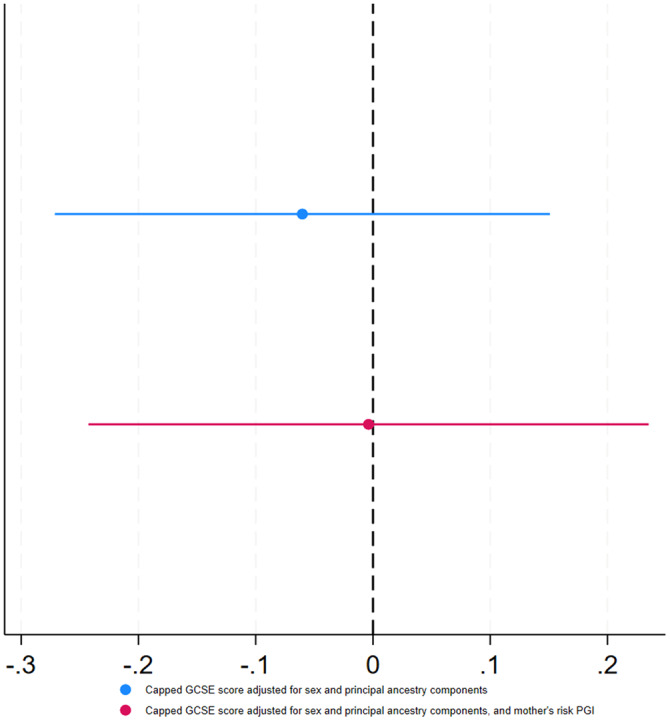
Association between the young person’s genetically instrumented MRB Index and their educational achievement. Error bars represent the 95% confidence intervals.

Figure 1 shows associations between the genetically instrumented MRB index and capped GCSE points score of young people. There was little evidence of an impact of the genetically instrumented MRB Index (F-statistic = 3.44) on capped GCSE score when adjusted for the sex and principal ancestry components ( $\hat{\beta }$ = −0.06, 95% CI: [−0.27,0.15]), or when additionally adjusted for the maternal risk PGI ($\hat{\beta }$ = 0.00, 95% CI: [−0.24,0.24]). The results for the binary outcome were similar; there was little evidence that risk behaviours influenced educational achievement adjusted for maternal risk PGI ($\hat{\beta }$ = −0.02, 95% CI: [−0.14, 0.10]) (Supplementary Fig. 1).

**Fig. 2:**
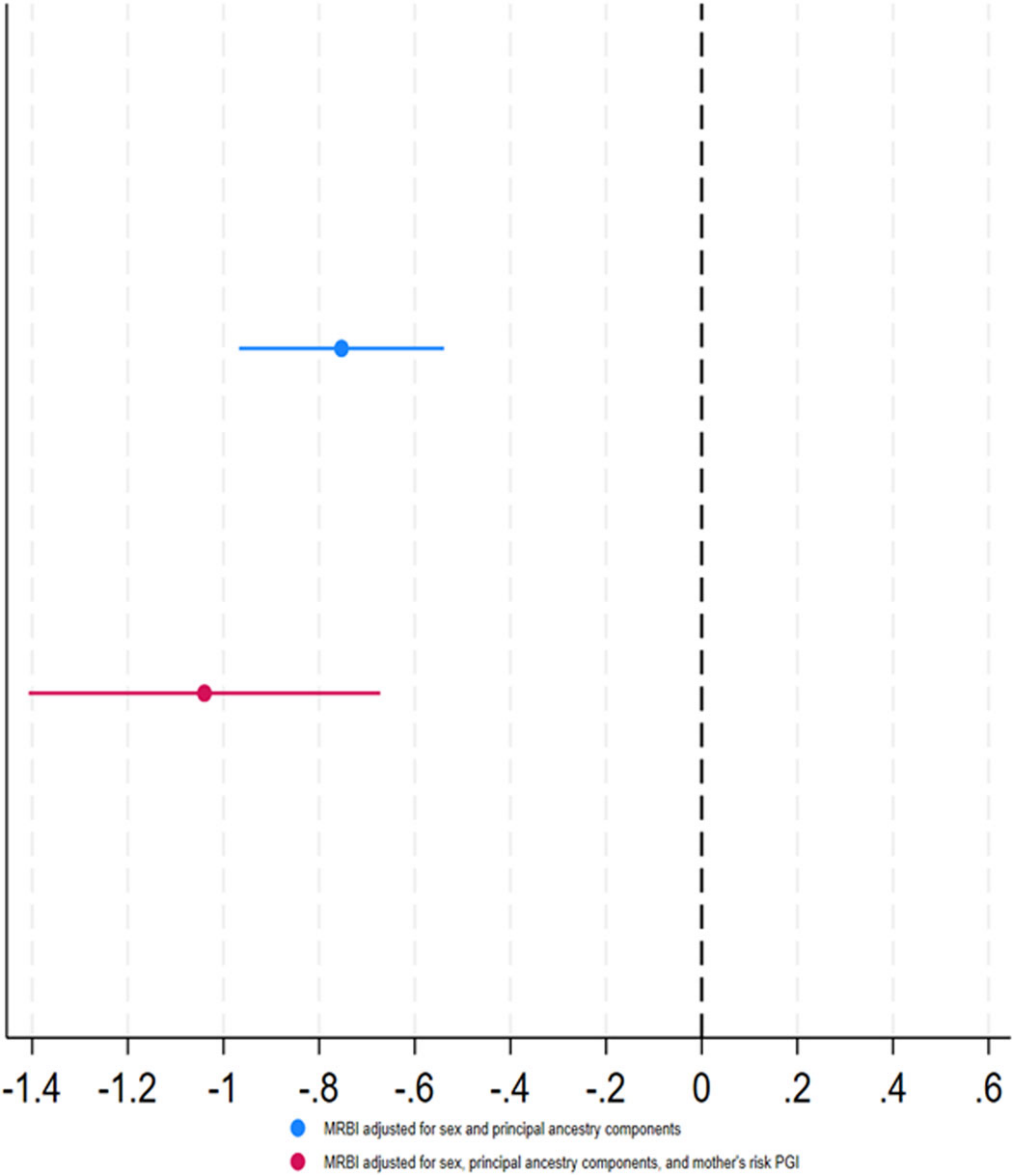
Association between young people’s genetically instrumented educational achievement (capped GCSE score, standardised) and their MRB index. Error bars represent the 95% confidence intervals.

Figure 2 shows the association between the genetically instrumented capped GCSE score and the MRB index of young people. There was a negative association between genetically instrumented education (F-statistic = 725.58) and MRB index ($\hat{\beta }$ = −0.75, 95% CI: [−0.97, −0.54]) when adjusting for the sex and principal components of ancestry and when additionally adjusting for the mother’s education PGI ($\hat{\beta }$ = −1.04, 95% CI: [−1.41, −0.67]). Attenuation with adjustment for the mother’s education PGI were similar for the binary outcome (Supplementary Fig. 2).

## Discussion

In a cohort of adolescents, an index of multiple risk behaviours was phenotypically associated with educational achievement at 16 after adjustment for confounders. Genetic analysis using GREML indicated that both traits were heritable and shared genetic architecture, with considerable genetic overlap between the two traits. Consistent with the results of phenotypic models, genetic variation associated with risk behaviours was negatively associated with educational achievement. Furthermore, bidirectional MR suggested that educational achievement affects risk behaviours and that engagement in risk behaviours may be partly driven by an individual’s educational achievement or a closely related trait. In contrast, we found little evidence that genetic variation associated with engagement in risk behaviours causally affected educational achievement, but these estimates were less precise.

A possible explanation for these results is familial factors, such as indirect genetic effects of parents on their children. Indirect genetic effects can occur when the parents’ genetic variants affect the offspring through environmental mechanisms (i.e. not via direct genetic transmission). For example, ref. 16 found that parents’ non-transmitted polygenic indexes were associated with the educational achievement of their children 29.9% as strongly (*p* = 1.6 × 10^−14^) as parents transmitted polygenic indexes^17^ This is consistent with results found in Howe et al.’s (2022) within-sibship GWAS, where the association of genetic variants with educational attainment and phenotypes from population estimates, such as BMI and smoking, may be inflated by indirect genetic effects. However, adjusting our analysis for mothers’ polygenic indexes only modestly attenuated the effects. Additional data is needed to investigate how indirect genetic effects influence these relationships in genotyped mother–father–child trios^18^

The MRB index had a negative phenotypic association with educational achievement for both achievement measures. We showed a decrease in the capped GCSE score of 0.14 SD (95% CI: [−0.17, −0.12]) per SD higher engagement in risky behaviours; these results were slightly attenuated in the full model when controlled for confounders. The fully adjusted model showed a negative association in the capped GCSE score of 0.12 SD (95% CI: [−0.14, −0.10]). Similar results were observed when exploring the association between the MRB index and the probability of gaining five A*-C grades at GCSE, including in English and Mathematics. These results are consistent with previous results based on the ALSPAC cohort, where multiple risk behaviours were negatively associated with education achievement, presenting a reduction in test scores of 6.31 points (95% CI: [−7.03, −5.58])^4^

Our estimates of the heritability of educational achievement are in line with those reported by previous studies. Among many others, ref. 19 estimated heritability for educational outcomes of 0.21 for GCSE Mathematics, 0.15 for GCSE English and 0.17 for GCSE Science. Likewise, ref. 20 estimated heritability of reading performance of 0.38 in a genetic study using the Western Reserve Reading Project data in Ohio, USA. Krapohl and Plomin^21^ estimated heritability of educational attainment of 0.31 in their study of socioeconomic position and offspring education. Our results from bivariate GREML also indicate that engagement in risk behaviours had a strong negative genetic association with educational outcomes at 16 years, with a genetic correlation of −0.51 (95%CI: −1.04, 0.02) for our capped GCSE score and −0.82 (95%CI: −1.68, 0.04) for attaining 5 or more A*-C grades in Mathematics and English.

Our MR results provided little evidence that risk behaviours affected educational achievement ($\hat{{{{{{\rm{\beta }}}}}}}$ = −0.06, 95% CI: [−0.27,0.15]), with or without adjustment for the maternal risk PGI. In contrast, there was evidence of a causal effect of educational attainment on engagement in risk behaviours ($\hat{{{{{{\rm{\beta }}}}}}}$ = −0.75, 95% CI: [−0.97, −0.54]). This may be because the MR estimate of the effect of education on risk behaviours was considerably more precise, reflecting an educational attainment PGI which was a much stronger instrument than the PGI for risk behaviours.

The risk behaviour literature shows that the risk behaviours that we considered frequently co-occur and tend to cluster during adolescence^22,23^. Existing studies investigating clustered risk behaviours focus only on small subsets of behaviours, such as alcohol use and smoking^24^, failing to account for behaviours such as self-harm and criminal or delinquent behaviour. We consider a wider range of clustered risk behaviours that allows us to capture risk associations with education more comprehensively. While we had insufficient power to draw firm conclusions about the effects of risk behaviours on educational attainment, our results do imply that educational achievement, or a closely related trait, affects risk behaviours. This supports current literature indicating that universal school-based interventions to improve students’ outcomes may have reduced the rates of risk behaviours^25^. Findings therefore suggest that these interventions could improve student outcomes and lessen the burden on public health services whilst reducing adolescent risk behaviours.

However, there are some limitations to our analysis. Missing data on risk behaviours and confounders reduced power (especially for GCTA analysis, which did not use imputed data) and may have introduced bias. Likewise, although the multiple risk behaviour index comprised a wide range of behaviours, by assigning each risk behaviour the same weight, we assumed that all risk behaviours contribute equally to associations with educational achievement. Horizontal pleiotropy might also have affected our results if genetic variants for educational attainment also affect other traits influencing risk behaviour. It is challenging to investigate further as most pleiotropy robust methods require GWAS summary data rather than individual-level data as used in this study. Future work could, however, employ multivariate Mendelian Randomisation^26^ to study the direct effect of risk behaviour and educational achievement^27^. The lack of genetic data on fathers meant we could not adjust for paternal genotype, and indirect genetic effects involving fathers might have influenced our results. However, controlling for maternal genotype only modestly attenuated associations, suggesting that indirect genetic effects were unlikely to explain our findings fully. Nevertheless, assessment of these relationships using well-powered familial analysis, like M-GCTA^26^ and bigger samples, could shed more light on passive environmental confounding or indirect genetic effects, leading to a better understanding of causation. Furthermore, some of the risk behaviours were measured via questionnaires, which may have introduced recall and desirability biases, where participants might have underreported socially perceived undesirable behaviours. Future work could investigate whether some risk behaviours are more closely linked to education than others. Our study only investigated the association of these phenotypes with common genetic variation, and future studies could investigate the impact of rare genetic variation.

In summary, we explored the genetic architecture of risk behaviour engagement in educational achievement and the bidirectional causal effect of these traits. We found evidence that higher educational achievement, or a closely related trait, will likely reduce risk behaviours. However, we found little evidence that risk behaviours affected educational achievement, although statistical power was limited. Our results add to existing evidence that educational achievement may be an effective intervention target for risky behaviours.

## Methods

### Study participants

The Avon Longitudinal Study of Parents and Children (ALSPAC) is a prospective birth cohort based in the Bristol and Avon area in the UK. ALSPAC invited pregnant women to participate if they were residents in the area and had expected delivery dates from 1st April 1991 to 31st December 1992. From 14,541 pregnancies initially enrolled, 13,988 children were alive at 1 year of age. When the oldest children were approximately seven, the study attempted to include eligible cases who did not originally participate in the study. The total sample size for analyses using any data collected after the age of seven is 15,447 pregnancies, resulting in 15,658 foetuses. Of these, 14,901 children were alive at one year of age. Details of the enrolment phases are provided elsewhere^28–30^ Consent for biological samples was collected per the Human Tissue Act (2004) (for full information on ALSPAC ethical approval, please see:  http://www.bristol.ac.uk/alspac/researchers/research-ethics/). Informed consent for the use of data collected via questionnaires and clinics was obtained from participants following the recommendations of the ALSPAC Ethics and Law Committee at the time. Ethical approval for the study was obtained from the ALSPAC Law and Ethics Committee and local research ethics committees (NHS Haydock REC: 10/H1010/70). This study has been pre-registered with ALSPAC under proposal number B3557. Completion of individual questionnaires was taken as consent for the data from that questionnaire, with additional written permission from parents for the use of clinic data. At age 16, young people and their parents gave written informed consent for the use of the young person’s genetic information. At age 18, study children were sent ‘fair processing’ materials describing ALSPAC’s intended use of their health and administrative records. They were given clear means to consent or object via a written form. Education data were not extracted for participants who objected or were not sent fair processing materials^28,31^ This project was registered with ALSPAC under proposal number B3557. ALSPAC has a lower share of ethnic minority participants than the UK population but was otherwise broadly representative at baseline^29^. All ethical regulations relevant to human research participants were followed.

### Sample

**Fig. 3:**
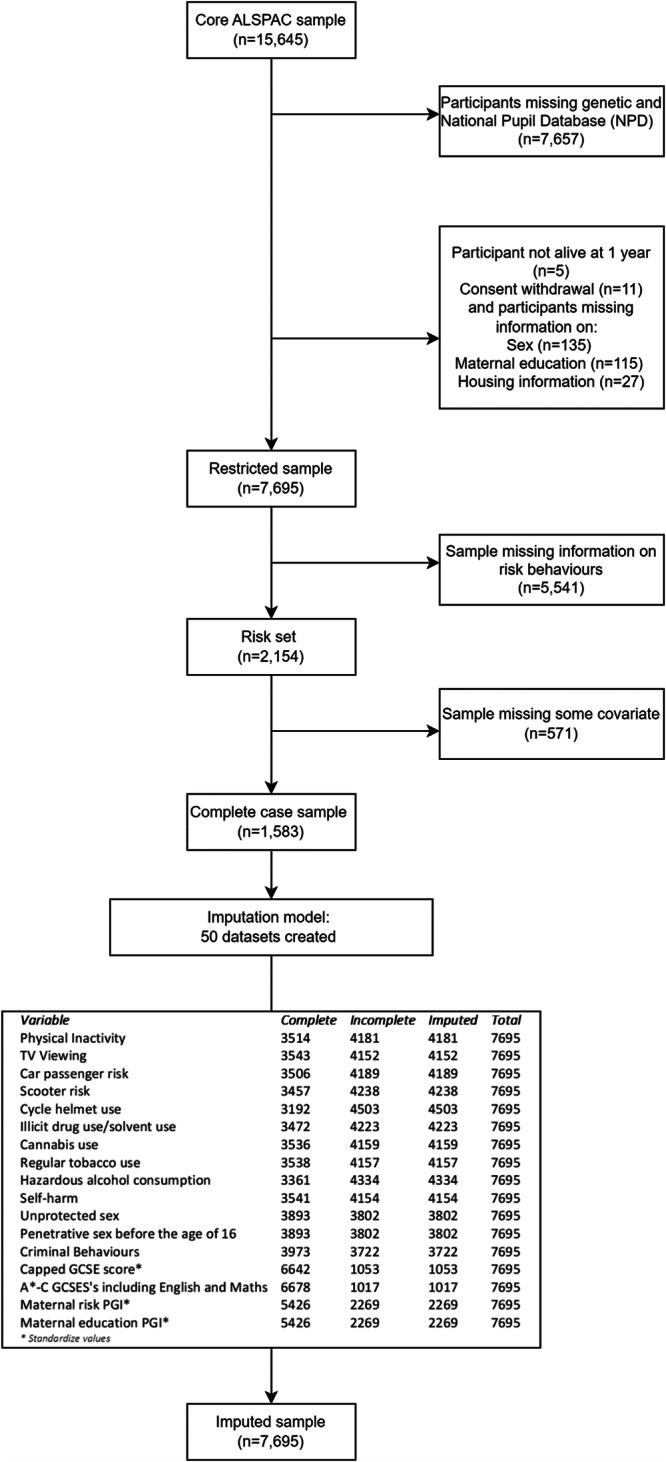
STROBE diagram. The diagram describes the selection of the complete case sample and the imputed sample.

Attrition and patterns of missingness across variables reduced the complete case analytical sample from 15,645 participants to 1583 (Fig. 3). Due to attrition, a substantial number of participants originally included in ALSPAC did not have genetic data or linkage to the National Pupil Database (NPD) (*N* = 7657). We further excluded consent withdrawals(*n* = 11), participants not alive at 1 year (*n* = 5), participants with no sex information (*n* = 135) and participants with no socioeconomic information available (maternal education (*n* = 115) and housing tenure (*n* = 27). Thus, we restricted the analytic sample to the 7695 participants alive at 1 yr with genetic and NPD data, sex, and socioeconomic information from infancy (maternal educational qualifications and housing tenure) who had not withdrawn consent. Within this sample, missing data in remaining variables (risk behaviours and other covariates) was imputed. We performed multiple imputation by chained equations^32^, with 50 imputed datasets created. We used the imputed dataset for phenotypic analyses and bidirectional MR. GREML analyses used the complete case sample of participants with genetic information and complete data on all exposures, outcomes, and covariates (*N* = 1583). We carried out the phenotypic analysis and MR in both the complete case sample and imputed datasets; we present results on the imputed sample in the main manuscript with complete case analyses given in the supplementary material (Supplementary Tables 3–7, 12–17 and supplementary Figs. 3, 4). For the imputation model, we included marital status, mother’s smoking status, maternal education, housing tenure and parental social class as auxiliary variables. We used logistic regression to impute the risk behaviours, linear and truncated regression for continuous variables and ordered logistic regression to impute categorical variables. Multiple imputation resulted in an imputed sample size of 7695.

### Genotyping

ALSPAC children were genotyped using the Illumina HumanHap550 platform, and standard quality control procedures were applied. Individuals were then excluded based on sex mismatch, minimal or excessive heterozygosity, disproportionate individual missingness (>3%) and insufficient sample replication (UBD <0.8). During genetic quality controls, individuals with non-European ancestry were removed, as is often done in genetic studies, to minimise bias introduced by ancestral population stratification. SNPS with a minor allele frequency is <1%, call rate of <95% or evidence of Hardy-Weinberg disequilibrium (*p*val <5 × 10^7^) were removed. Cryptic relatedness was measured as the proportion of identity by descent (IBD >0.1). Imputation was performed using impute v2.2.2 to the Haplotype Reference Consortium (HRC) panel, and SNPs with poor imputation quality (infoscore <0.08) removed.

### Measures

#### Multiple risk behaviours (MRBs) at age 16

An index of multiple risk behaviours (MRBs) was derived from two main data collections during the participants’ adolescence: a self-completed questionnaire issued during a clinic assessment at age 15 and a self-completed postal questionnaire at age 16. We coded 13 risk behaviours into binary format (no = 0; yes = 1) following ref. 4 and then calculated an MRB index as the total number of risk behaviours each participant had engaged in. The underlying risk behaviours that we used to construct the risk behaviour index were largely already dichotomised (6 out of the 13 risk behaviours). None of the underlying risk behaviours were continuous. This risk behaviour index has been previously used in the literature^4,10,33^. We further carried out extra analysis to check the consistency of the index; these results are available in the supplementary information (Supplementary Tables 8–11). We tested the internal consistency of the index based on the Cronbach alpha and Pearson’s correlations, and also carried out a factor analysis. The results based on an updated index excluding two items with the lowest item-test correlation, and using the first factor as the exposure, did not alter conclusions (Supplementary Tables 14–17 and Supplementary Figs. 5–8).

The study website contains details of available data through a searchable data dictionary and variable search tool: http://www.bristol.ac.uk/alspac/researchers/our-data/.

The risk behaviours included in the index were:

Physical inactivity: Participant has typically exercised <5 times per week over the past year.

TV viewing: Participant spent three or more hours watching TV on average daily across the week.

Car passenger risk: The participant had been in a car passenger at least once in their lifetime where the driver (1) had consumed alcohol, (2) did not have a valid licence, or (3) the participant chose not to wear a seat belt last time travelling in a car, van, or taxi.

Scooter risk: Participants reported that they had last ridden a scooter within the previous four weeks and had not used a helmet on the most recent occasions.

Cycle helmet use: If the participant reported that they had last ridden a bicycle within the previous 4 weeks and had not used a helmet on the most recent occasion.

Illicit drug use/solvent use: In the year since their 15th birthday, the participant had either been a regular user (used more than five times) of one or more illicit drugs (excluding cannabis), including amphetamines, ecstasy, lysergic acid diethylamide (LSD), cocaine, ketamine or inhalants including aerosols, gas, solvents, and poppers.

Cannabis use: Participants who reported using cannabis ‘sometime, but less often than once a week’ or more regular use were classified as occasional users.

Regular tobacco use: Participant has never smoked and is regularly smoking at least one cigarette per week.

Hazardous alcohol consumption: In the past year, participants had scored eight or more on the Alcohol Use Disorders Identification Test (AUDIT), indicating hazardous alcohol consumption.

Self-harm: Participant said they had purposely hurt themselves in some way in their lifetime.

Penetrative sex before the age of 16: Participant reported having had penetrative sex in the preceding year and that they were under 16 at the time.

Unprotected sex: Participant engaged in penetrative sex without using contraception on the last occasion they had had sex in the past year.

Criminal and delinquent behaviour: Participant reported that at least once in the past year, they had undertaken at least one of the following: carried a weapon; physically hurt someone on purpose; stolen something; sold illicit substances to another person; damaged property belonging to someone else either by using graffiti, setting fire to it, or destroying or damaging it in another fashion; subjected someone to verbal or physical racial abuse; or been rude/rowdy in a public place.

As each of the risk behaviours can be represented as a binary indicator (see Table 3 for descriptives of individual risk behaviours), we can denote the variable measuring engagement in risk behaviour *j* for each individual *i* by the binary indicator as follows:

$${w}_{{ij}}=\left\{\begin{array}{c}1{{{{{{\mathrm{if}}}}}}}\,{{{{{{\mathrm{individual}}}}}}}\,i\,{{{{{{\mathrm{engages}}}}}}}\,{{{{{{\mathrm{in}}}}}}}\,{{{{{{\mathrm{risk}}}}}}}\,{{{{{{\mathrm{behaviour}}}}}}}\,j,\\ 0{{{{{{\mathrm{otherwise}}}}}}}\end{array}\right.$$

**Table 3:** Descriptive statistics for educational achievement measures and MRB index in the imputed sample and individual multiple risk behaviours in the pre-imputed set

Continuous variables	*N*	Mean	SD	Min	Max
Capped GCSE score^a^	7695	331.19	90.16	−0.52	590.89
5 or more A*-C GCSE’s including English and Maths^b^	7695	0.57	0.49	0	1
MRB index	7695	3.48	2.18	0	12.08
Binary variables					
Multiple risk behaviours (MRBs)	*N*^c^		% Engaging		
Physical inactivity	3556		74%		
TV viewing	3584		21%		
Car passenger risk	3547		30%		
Scooter risk	3497		20%		
Cycle helmet use	3227		24%		
Illicit drug use/solvent use	3512		8%		
Cannabis use	3578		10%		
Regular tobacco use	3579		12%		
Hazardous alcohol consumption	3399		36%		
Self-harm	3582		19%		
Penetrative sex before the age of 16	3933		17%		
Unprotected sex	3933		3%		
Criminal and delinquent behaviours	4017		47%		

^a^Capped GCSE is a continuous measure of educational achievement.

^b^Achieved 5 or more is a binary measure of educational achievement.

^c^Pre-imputation sample analysis was restricted to unrelated ALSPAC participants with genetic data and linked GCSE records, alive at 1 year, who had not withdrawn consent, complete sex information and that had enough maternal socioeconomic information. Missing data were imputed using multiple imputation by chained equation.

Since we are looking at the overall engagement across a range of risk behaviours rather than individual effects of each, we then create a new single variable called the multiple risk behaviour index (MRBI), defined for each individual *i* as the sum of all behaviours, as follows:

$${{{{{{{\mathrm{MRBI}}}}}}}}_{i}=\mathop{\sum }\limits_{j=1}^{13}{w}_{{ij}}$$

The new regressor ${{{{{{{\mathrm{MRBI}}}}}}}}_{i}$ is our exposure of interest summarised in Table 3.

### Educational achievement

Information on educational achievement was obtained via record linkage to the National Pupil Database (NPD). Managed by the Department of Education in England, the NPD includes data collected from school students and higher education students from 2 to 21 years. This dataset comprises the most complete and accurate record of compulsory educational achievement available in England. Educational measures were based on participants’ General Certificate of Secondary Education (GCSE) qualifications, which are taken during educational Key Stage 4 when pupils are aged between 14 and 16 years old. At the time, Key Stage 4 marked the end of compulsory education in England. For this analysis, we used two measures of achievement. The first was the capped GCSE score, a continuous measure which sums the student’s eight best grades to obtain a measure of overall achievement commonly used in educational research. Individual GCSE qualifications in each subject contribute 58 points for an A* through to 16 points for a G and 0 for a U (ungraded). Our second measure of educational achievement was a binary indicator of whether participants achieved five or more A*-C grades at GCSEs. We used this as it is the qualification requirement for entry to many post-16 education and training courses.

### Polygenic indexes (PGI)

We used the largest existing genome-wide association studies (GWAS) to identify single-nucleotide polymorphisms (SNPs) associated with risk behaviours^34^ and educational achievement^35^. After sub-setting GWAS results for both phenotypes to SNPs that were available in ALSPAC, we used the MRInstruments R package to identify SNPs which were independently associated (at *p* < 5 × 10^−8^) with the phenotypes (clumping parameters: R2 = 0.01, 10,000 kb). This resulted in 303 SNPs associated with risk behaviour and 3952 SNPs associated with educational achievement. PGIs based on these SNPs were then derived in PLINK 1.9 by summing trait-increasing alleles. SNPs were weighted by each allele’s regression coefficient from the GWAS so that genetic variants with greater effect contributed more to the scores. Finally, scores were standardised for analysis. The children’s educational achievement PGI explained 9.83% of the variation in the capped GCSE score (continuous outcome), while the children’s risk behaviour PGI explained 0.05% of the variation in the MRB index. The mother’s educational achievement PGI explained 6.94% of children’s capped GCSE scores, and the mothers' risk behaviour PGI explained 0.16% of the variation in children’s risk behaviours.

### Statistical analysis

In order to explore the association between the MRB index and educational achievement, we carried out three types of analyses. First, we examined phenotypic associations between the MRB index and the continuous and binary measures for educational achievement in the ALSPAC cohort. Secondly, to explore the genetic underpinnings of engagement in risk behaviour and educational achievement, we performed univariate GREML to estimate the heritability of both traits, and bivariate GREML to explore the genetic correlation of these behaviours. GREML analysis was carried out in the complete case sample, as GREML cannot be readily performed using multiply imputed phenotype data. Third, given the possible confounding bias which can affect estimates based on observational data, we used bidirectional MR analyses to estimate causal associations between the MRB index and educational measures in our imputed datasets. Below we expand on these analytical methods.

#### Phenotypic associations

We used linear and logistic regression to estimate the association of the MRB Index with capped GCSE score (continuous outcome) and gaining five or more GCSE grade A*-C (binary outcome). Base models adjusted for the young person’s sex. Since other factors may confound the association of educational achievement and the number of risk behaviours, we also estimated these associations adjusted for the following potential socioeconomic confounders: parental social class, maternal education, and housing tenure at the time of the child’s birth. Lastly, we estimated a third set of associations adjusted for the child’s cognitive ability. Table 4 shows the summary statistics for these variables in the imputed sample (see supplementary Tables 1, 2 for the complete case sample).

**Table 4:** Summary statistics of parental social class, housing tenure, maternal education, and participants’ sex

Variables	*N*	%
Parental social class (*N* = 7695)		
Professional	1113	14.5
Managerial and technical	3316	43.4
Skilled non-manual	1929	25.2
Skilled manual	923	12
Partially unskilled	365	4.74
Unskilled	48	0.63
Housing tenure (*N* = 7695)		
Owned	6127	79.6
Council rented	782	10.2
Privately rented	786	10.2
Maternal education (*N* = 7695)		
<O level	1943	25.3
O level	2674	34.8
A level	1897	24.7
Degree	1182	15.4
Participant’s Sex (*N* = 7695)		
Female	3945	51.3
Male	3750	48.7

#### Genotypic associations

We conducted genomic-based restricted maximum likelihood (GREML) to examine the genetic overlap between the MRB Index and educational achievement. These models were carried out using Genome-wide Trait Analysis (GCTA)^36^. GCTA uses a genomic restricted maximum likelihood (GREML) method to estimate the proportion of phenotypic variance that can be statistically explained by all measured genome-wide single-nucleotide polymorphisms (SNPs), known as the SNP-based heritability. GCTA estimates heritability by comparing the genetic similarity of unrelated individuals to their phenotypic similarities. Unrelated participants (defined as more distantly related than second cousins) were determined using Genetic Relatedness Matrices (GRMs)^36^ If a phenotype can be (in part) explained by genetic variation, then we would expect more genetically similar individuals to be more phenotypically similar^37^. We first estimated univariate models to test the SNP heritability of the educational outcomes and MRB index, specified as:

$$y=X\beta +g+\varepsilon$$

where $y$ is the phenotype, $X$ is a series of covariates, $g$ is a normally distributed random effect with variance ${\sigma }_{g}^{2}$ and $\varepsilon$ is a residual error with variance ${\sigma }_{\epsilon }^{2}$. The SNP-based heritability can then be estimated as the proportion of total phenotypic variance that is attributable to a genotypic variance of the phenotype:

$${h}_{{{{{{{\mathrm{SNP}}}}}}}\,}^{2}=\frac{{\sigma }_{g}^{2}}{{\sigma }_{g}^{2}+{\sigma }_{\epsilon }^{2}}.$$

To control for differences between ancestral populations in allele distributions which could potentially bias the estimate, the first 20 principal components of inferred population structure were included in the analyses as covariates.

We estimated genetic correlations between the MRB Index and both measures of educational achievement using bivariate GCTA^38^. Genetic correlations allow us to quantify the overlap in SNPs associated with multiple phenotypes. Specifically for this study, the genetic correlation shows the proportion of the phenotypic correlation between the MRB index and education that is explained by common variation. Genetic correlations are estimated as:

$${r}_{g}=\frac{{{{{{{\mathrm{co}}}}}}}{{{{{{\mathrm{v}}}}}}}_{g}(A,B)}{\sqrt{{{{{{{\mathrm{va}}}}}}}{{{{{{\mathrm{r}}}}}}}_{g}\left(A\right){{{{{{\mathrm{va}}}}}}}{{{{{{\mathrm{r}}}}}}}_{g}(B)}}$$

where ${r}_{g}$ is the genetic correlation between phenotypes $A$ and $B$, ${{{{{{\mathrm{va}}}}}}}{{{{{{\mathrm{r}}}}}}}_{g}(A)$ is the genetic variance of phenotype $A$, and ${{{{{{\mathrm{co}}}}}}}{{{{{{\mathrm{v}}}}}}}_{g}(A,B)$ is the genetic covariance between phenotypes ${A}$ and $B$. Genetic correlations reflect common genetic architecture, where two phenotypes are influenced by the same SNPs. GCTA does not support GREML using multiply imputed phenotype data, so these analyses were performed in the subset of the analytic sample who had complete phenotypic information (*N* = 1735).

#### Bidirectional Mendelian randomisation (MR)

Mendelian randomisation (MR) is a statistical method which can evaluate causal effects between purported exposures and outcomes in observational data by using genetic variants as instrumental variables for exposures. MR relies on the random assortment of alleles from parents to children which occurs during gamete formation and conception^39^. Since the genetic variants associated with the exposure do not change in response to a person’s health or environmental circumstances, associations between exposure-associated genetic variants and the outcome are not affected by classical confounding or reverse causation, which often affects estimates from observational studies^40^. For MR estimates to be valid, the genetic instruments must meet three assumptions: (1) relevance, it must associate with the exposure, (2) independence, there must be nothing that causes both the instrument and the outcome, and (3) exclusion, the association of the instrument and the outcome must be entirely mediated via the exposure^41^ We tested the first assumption using partial F-statistics.

**Fig. 4:**
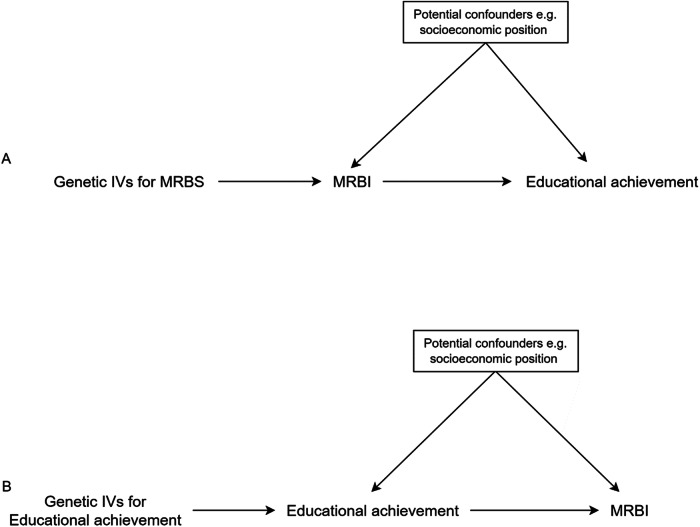
Directed acyclic graph of a bidirectional MR presenting the relationship between the MRB Index and educational achievement. Panel **A** depicts the relationship between the MRB index and educational achievement, while Panel **B** illustrates the bidirectional association between educational achievement and the MRB index. PGI refers to polygenic index and MRBI stands for multiple risk behaviour index.

For educational and risk behaviours, a causal effect in either direction is plausible, so we used bidirectional MR. Bidirectional MR is an extension of a standard MR analysis which attempts to differentiate whether the exposure is a cause of the outcome, a consequence of the outcome, or if there is a true bidirectional causal effect between them (Fig. 4)^42^

First, we used MR to estimate the effect of educational achievement on risk behaviours. We used a two-stage least squares instrumental variable model (Stata’s ivreg2) with the risk behaviours index as the outcome and instrumented educational achievement using a polygenic index of SNPs previously associated with years of schooling^35^ Next, we used MR to estimate the effect of risk behaviours on educational achievement by reversing the outcome and exposure. In this second analysis, the capped GCSE points score was the outcome, and we instrumented the risk behaviours index using a polygenic index of SNPs previously associated with risk-taking behaviour^34^. For each outcome, two sets of models were run: one which adjusted for the young person’s sex and their first 20 principal components of ancestry, and a model which also adjusted for factors associated with maternal genotype by including the mother’s polygenic index. Likewise, for the binary outcome of obtaining five or more A*-C GCSEs, we used a two-stage least squares instrumental variable model, and again instrumented the risk behaviours index using a polygenic index of SNPs previously associated with risk-taking behaviour.

### Reporting summary

Further information on research design is available in the Nature Portfolio Reporting Summary linked to this article.

### Supplementary information

Peer Review File

Supplementary material

Reporting Summary

## Data Availability

The informed consent obtained from ALSPAC participants does not allow the data to be made freely available through any third-party maintained public repository. Data used for this submission can be made available on request to the ALSPAC Executive. The ALSPAC data management plan describes in detail the policy regarding data sharing, which is through a system of managed open access. Full instructions for applying for data access can be found here: http://www.bristol.ac.uk/alspac/researchers/access/. The GWAS summary statistics for both risk behaviours and educational attainment used in the analyses are available through the Social Science Genetic Association Consortium (SSGAC). Available through the SSGAC website: https://www.thessgac.org/.

## Code availability

All the code used to clean and analyse the data for this study is available: https://github.com/MichelleSpano/Risk-behaviours
