# Supplementary material for: Genetic associations of risk behaviours and educational achievement
Source: Commun Biol. 2024 Apr 10;7:435. doi: 10.1038/s42003-024-06091-y (PMC11006670; doi:10.1038/s42003-024-06091-y)

Supplementary

Contents:

Supplementary Table 1: Descriptive statistics for MRBs in complete case sample.

Supplementary Table 2: Descriptive statistics for educational measures and MRB Index in complete case sample.

Supplementary Table 3: Comparison of observed characteristics between participants in the complete dataset (N=1,583) and incomplete dataset (N=14,062).

Supplementary Table 4: Association of capped GCSE score and score with an index of multiple risk behaviours, based on the complete case sample.

Supplementary Table 5: Association of achieving five or more A*-C GCSE score with an index of multiple risk behaviours, based on the complete case sample.

Supplementary Table 6: Associations of probability of gaining 5 or more A*-C GCSEs including English and Maths with an index of multiple risk behaviours, based on imputed data (N=7,695).

Supplementary Table 7: Associations of capped GCSE score with an index of multiple risk behaviours, based on imputed data (N=7,695)

Supplementary Figure 1: Association between the young person's genetically instrumented multiple risk behaviours (MRB) index and the probability of gaining 5 or more A* - C GCSEs including English and Maths.

Supplementary Figure 2: Association between the young person's genetically-instrumented educational attainment (obtaining attaining five or more A*-C GCSEs including English and Maths), and their Multiple Risk Behaviours (MRB) Index.

MRB Index consistency analyses

Supplementary Table 8: Cronbach alpha item analysis of the 13 risk behaviours.

Supplementary Table 9: Cronbach alpha item analysis, excluding physical inactivity and TV viewing.

Supplementary Table 10: Pearson's correlations coefficient.

Supplementary Table 11: Factor analysis of the 13 risk behaviours.

Phenotypic analyses

Supplementary Table 12: Associations of capped GCSE score with individual risk behaviours, based on complete case sample.

Supplementary Table 13: Associations of capped GCSE score with an index of the 13 original multiple risk behaviours, based on the complete case sample.

Supplementary Table 14: Associations of capped GCSE score with an index of 11 risk behaviour that were highly correlated with the overall MRB Index scale.

Supplementary Table 15: Associations of capped GCSE score with first factor, based on complete case sample.

GREML

Supplementary Table 16: Univariate model including for both MRB indexes, educational achievement measures, and individual risk behaviours.

Supplementary Table 17: Bivariate estimates of educational achievement, different risk behaviours measures and first factor.

MR

Supplementary Figure 3: Association between the young person's genetically-instrumented MRB Index (13 behaviours) and their educational achievement (capped GCSE score, standardised).

Supplementary Figure 4: Association between the young person's genetically-instrumented educational achievement (capped GCSE score, standardised), and their MRB Index (13 behaviours).

Supplementary Figure 5: Association between the young person's genetically-instrumented MRB Index (11 behaviours) and their educational achievement (capped GCSE points score, standardised).

Supplementary Figure 6: Association between the young person's genetically-instrumented educational achievement (capped GCSE score, standardised), and their MRB Index (11 behaviours)

Supplementary Figure 7: Association between the first factor of the young person's genetically-instrumented risk behaviours and their educational achievement (GCSE capped points score, standardised).

Supplementary Figure 8: Association between the young person's genetically-instrumented educational attainment (capped GCSE score, standardised), and the MRB Index first factor.

Supplementary Table 1: Descriptive statistics for MRB in complete case sample.

**Multiple Risk Behaviours (MRB)**	**n**	**% Engaging**
Physical inactivity	3556	74%
TV viewing	3584	21%
Car passenger risk	3547	30%
Scooter risk	3497	20%
Cycle helmet use	3227	24%
Illicit drug use/solvent use	3512	8%
Cannabis use	3578	10%
Regular tobacco use	3579	12%
Hazardous alcohol consumption	3399	36%
Self-harm	3582	19%
Penetrative sex before the age of 16	3933	17%
Unprotected sex	3933	3%
Criminal and delinquent behaviours	4017	47%

Supplementary Table 2: Descriptive statistics for educational measures and MRB Index in complete case sample.

Variable	N	Mean	SD	Min	Max
Capped GCSE score	6654	329.67	89.56	0	540
A*-C GCSES's including English and Maths	6695	0.57	0.50	0	1
MRB Index	2171	3.19	1.97	0	11

Supplementary Table 3: Comparison of observed characteristics between participants in the complete dataset (N=1,583) and incomplete dataset (N=14,062)

	**Complete dataset N=1,583**	**Incomplete dataset N=14,062**	**Test for difference**
Continuous variables	***Mean***	***Mean***	***T-test***
MRB Index	3.18	3.17	-0.15
Capped GCSE score	375.28	304.06	-31.58
Maternal age	29.72	27.71	-16.95
Cognitive ability	109.09	102.05	-16.51
Categorical variables	***%***	***%***	***Chi²***
Achieved 5 or more A*- C GCSEs including English and maths			739.55
No	21.66	54.86	
Yes	78.34	45.14	
Sex			76.73
Male	42.02	52.54	
Female	57.98	47.46	
Maternal Education			407.52
<O level	13.86	33.18	
O level	34.22	34.72	
A level	30.92	20.75	
Degree	21.01	11.35	
Housing Tenure			343.38
Mortgage/owned	89.94	70.28	
Council rented	3.90	16.20	
Private/other rented	6.15	13.52	
Parent social class			199.34
Professional	19.06	12.10	
Managerial and technical	48.27	40.41	
Skilled non-manual	22.46	26.12	
Skilled manual	10.21	21.37	

Supplementary Table 4: Association of capped GCSE score and score with MRB index, based on the complete case sample.

Capped GCSE score	Model 1<br>N = 1877	Model 2<br>N=1711	Model 3<br>N=1693	Model 4<br>N=1583
		95% confidence intervals in brackets		
**MRB Index**	-0.07 [-0.09, -0.05]	-0.06 [-0.07, -0.04]	-0.06 [-0.07, -0.04]	-0.06 [-0.07, -0.04]
**Parental socioeconomic Position**		-0.14[-0.18, -0.11]	-0.13[-0.17, -10]	-0.10[-0.13, -0.07]
**Maternal Education ref: <O level**		0.18[0.14,0.21]	0.18[0.14,0.21]	0.08[0.05,0.11]
**Sex ref: male**		0.12[0.07,0.18]	0.12[0.07,0.18]	0.16[0.12,0.22]
**Housing tenure ref: Owned**			-0.10[-0.17, -0.04]	-0.09[-0.14, -0.03]
**Cognitive ability**				0.02[0.02,0.02]

Supplementary Table 5: Association of achieving five or more A*-C GCSE score with an index of multiple risk behaviours, based on the complete case sample.

A*-C GCSE's including English and Maths	Model 1<br>N=1877	Model 2<br>N=1711	Model 3<br>N=1693	Model 4<br>N=1583
		Exponentiated Coefficients. 95% confidence intervals in brackets		
**MRB Index**	0.86 [0.82, 0.91]	0.87 [0.82, 0.91]	0.83 [0.82, 0.92]	0.86 [0.81, 0.91]
**Parental socioeconomic Position**		0.70[0.61,0.80]	0.73[0.64,0.83]	0.76[0.66,0.88]
**Maternal Education ref: <O level**		1.34[1.17,1.54]	1.36[1.19,1.56]	1.06[0.91,1.24]
**Sex ref: male**		1.36[1.07,1.72]	1.37[1.08,1.73]	1.58[1.22,2.05]
**Housing tenure ref: Owned**			0.79[0.64,0.97]	0.83[0.66,1.05]
**Cognitive ability**				1.06[1.05,1.07]

Supplementary Table 6: Associations of probability of gaining 5 or more A*-C GCSEs including English and Maths with an index of multiple risk behaviours, based on imputed data (N=7,695)

A*-C GCSEs including English and Maths	Model 1	Model 2	Model 3	Model 4
		Exponentiated Coefficients. 95% confidence intervals in brackets		
**MRB Index**	0.81 [0.78, 0.84]	0.82[0.79, 0.86]	0.83 [0.80, 0.86]	0.81 [0.77, 0.84]
**Parental socioeconomic Position**		0.70[0.66, -074]	0.73[0.69,0.78	0.81[0.77,0.87]
**Maternal Education ref: <O level**		1.67[1.57,1.78]	1.65[1.55,1.77]	1.29[1.20,1.40]
**Sex ref: male**		1.74[1.56,1.58]	1.75[1.56,1.96]	2.00[1.76,2.27]
**Housing tenure ref: Owned**			0.69[0.62,0.75]	0.73[0.66,0.81]
**Cognitive ability**				1.07[1.07,1.08]

Supplementary Table 7: Associations of capped GCSE score with an index of multiple risk behaviours, based on imputed data (N=7,695)

Capped GCSE score	Model 1	Model 2	Model 3	Model 4
			95% confidence intervals in brackets	
**MRB Index**	-0.14 [-0.17, -0.12]	-0.12 [-0.14, -0.10]	-0.12 [-0.13, -0.10]	-0.11[-0.13, -0.09]
**Parental socioeconomic Position**		-0.19 [-0.22, -0.17]	-0.17 [-0.10, -0.14]	-0.10 [-0.12, -0.07]
**Maternal Education ref: <O level**		0.27 [0.24,0.29]	0.27 [0.24,0.29]	0.11[0.09,0.14]
**Sex ref: male**		0.30 [0.26, 0.34]	0.30 [0.26,0.34]	0.30[0.27,0.34]
**Housing tenure ref: Owned**			-0.18[-0.23, -0.15]	0.13[-0.17, -0.10]
**Cognitive ability**				0.03[0.03,0.03]

Supplementary Figure 1: Association between the young person's genetically instrumented multiple risk behaviours (MRB) index and the probability of gaining 5 or more A* - C GCSEs including English and Maths.

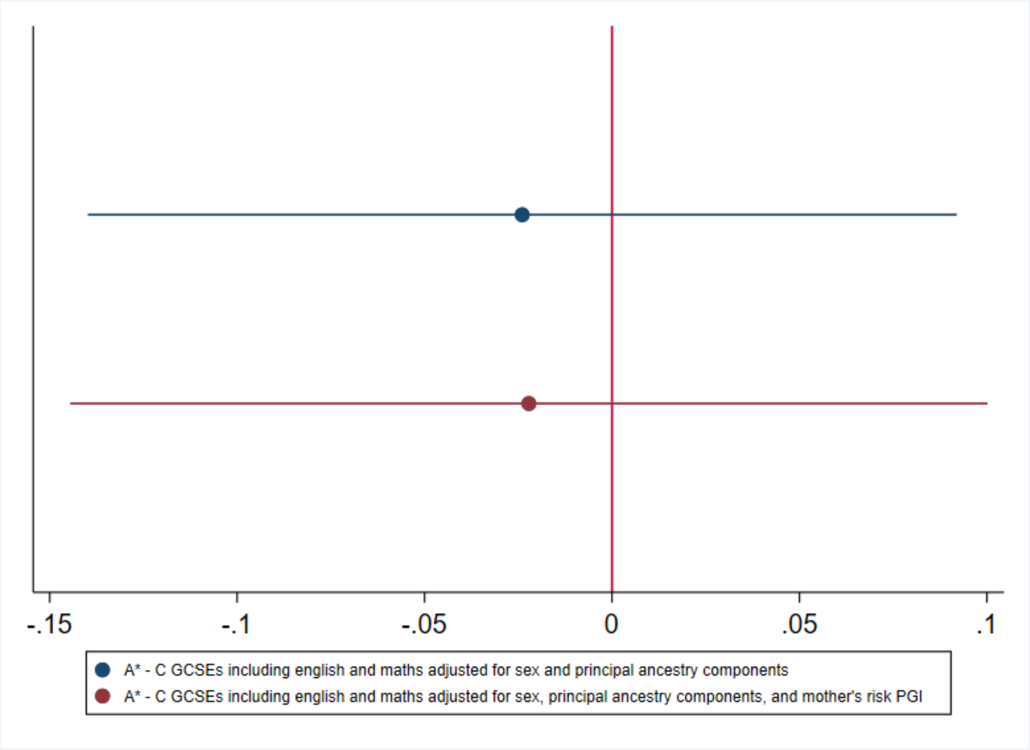

Supplementary Figure 2: Association between the young person's genetically-instrumented educational attainment (obtaining attaining five or more A*-C GCSEs including English and Maths), and their Multiple Risk Behaviours (MRB) Index.

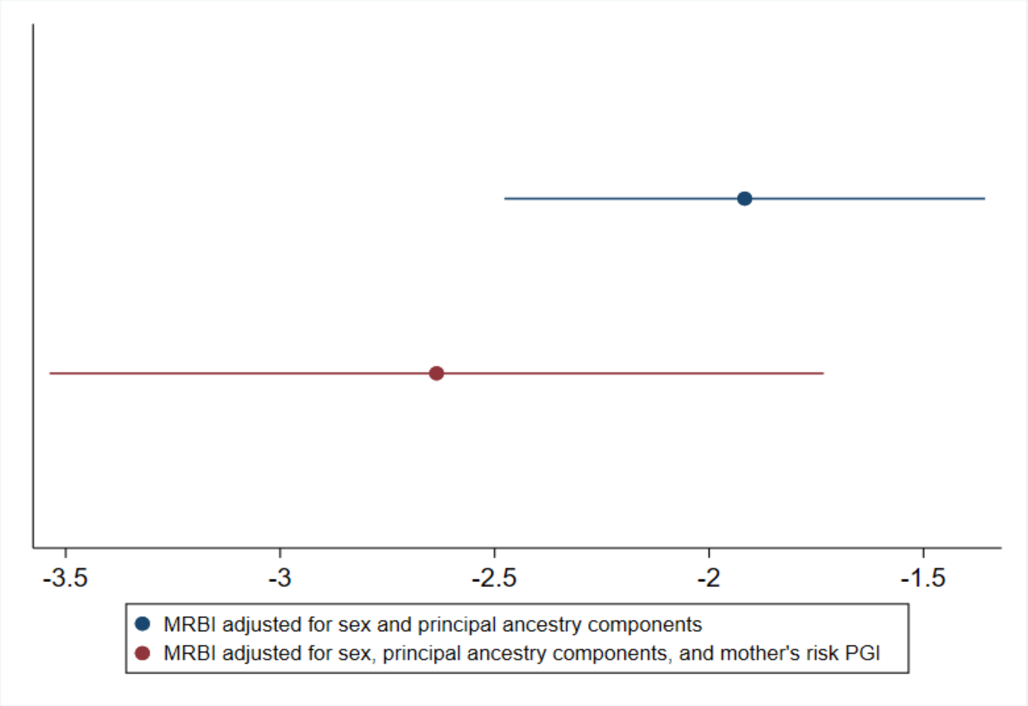

MRB Index consistency analyses

Supplementary Table 8: Cronbach alpha item analysis of the 13 risk behaviours.

Individual Risk Behaviours	n	Item-test correlation [a]	Item-rest correlation [b]	α [c]
Physical Inactivity	3514	0.25	0.04	0.65
TV viewing	3543	0.23	0.03	0.65
Car passenger risk	3506	0.53	0.34	0.59
Cycle helmet use	3192	0.41	0.21	0.61
Scooter risk	2457	0.27	0.18	0.62
Illicit drug use/solvent use	3472	0.53	0.42	0.59
Cannabis use	3536	0.52	0.39	0.59
Regular tobacco use	3538	0.57	0.44	0.58
Hazardous alcohol consumption	3361	0.58	0.39	0.58
Self-harm	3541	0.41	0.23	0.61
Penetrative sex before the age of 16	3893	0.57	0.32	0.60
Unprotected sex	3893	0.37	0.24	0.61
Criminal and delinquent Behaviour	3973	0.63	0.29	0.59
**Test scale**				0.63

[a] Item-test correlation shows how correlated the item is with the overall scale
[b] Item-rest correlation represents how correlated the item is with the other 12 items
[c] α shows the changes in the scale if the item was not included, if the alpha increases, then removing the item would improve the consistency of the measure.

Supplementary Table 9: Cronbach alpha item analysis, excluding physical inactivity and TV viewing.

	Number of Items	α
**MRB Index (13 behaviours) ***	13	0.63
**MRB Index (11 behaviours) ****	11	0.68

*Complete MRB Index including original 13 risk behaviours
**New MRB Index excluding activity and tv time

Supplementary Table 10: Pearson's correlations coefficients.

Risk Behaviour	*Physical inactivity*	*TV viewing*	*Car passenger risk*	*Cycle helmet use*	*Scooter risk*	*Illicit drug use/solvent use*	*Cannabis use*	*Regular tobacco use*	*Hazardous alcohol consumption*	*Self-harm*	*Penetrative sex before the age of 16*	*Unprotected sex*	*Criminal and delinquent behaviour*
Physical inactivity	1												
TV viewing	0.032	1											
Car passenger risk	0.0127	0.0284	1										
Cycle helmet use	-0.0485	-0.063	-0.1341	1									
Scooter risk	-0.0744	-0.0126	0.1643	-0.0955	1								
Illicit drug use/solvent use	0.0363	0.0252	0.2053	-0.0804	0.1279	1							
Cannabis use	0.019	-0.0376	0.2181	-0.0851	0.1426	0.5141	1						
Regular tobacco use	0.0584	-0.005	0.223	-0.1225	0.1608	0.4047	0.4059	1					
Hazardous alcohol consumption	0.0122	0.0054	0.269	-0.146	0.1447	0.2619	0.2788	0.2753	1				
Self-harm	0.0834	-0.0186	0.1404	-0.0572	-0.0184	0.1716	0.1441	0.196	0.1597	1			
Penetrative sex before the age of 16	0.0115	0.0276	0.14	-0.1208	0.0999	0.1485	0.1133	0.186	0.1454	0.1033	1		
Unprotected sex	0.0278	0.0221	0.0633	-0.0286	0.012	0.0698	0.0685	0.0938	0.0763	0.1215	0.3777	1	
Criminal and delinquent behaviour	-0.031	0.0058	0.1923	-0.1422	0.1869	0.2194	0.2335	0.237	0.2799	0.1665	0.2324	0.1122	1

Supplementary Table 11: Factor analysis of the 13 risk behaviours.

**Factors**	**Eigenvalue**	**Difference**	**Proportion**
Factor 1	2.08	1.59	0.99
Factor 2	0.49	0.21	0.23
Factor 3	0.28	0.12	0.13
Factor 4	0.15	0.10	0.07
Factor 5	0.06	0.05	0.03
Factor 6	0.01	0.05	0.00
Factor 7	-0.04	0.04	-0.02
Factor 8	-0.09	0.02	-0.04
Factor 9	-0.10	0.02	-0.05
Factor 10	-0.12	0.04	-0.06
Factor 11	-0.16	0.04	-0.08
Factor 12	-0.19	0.07	-0.09
Factor 13	-0.26	.	-0.12
**Chi^2**	3142.64	**Prob>Chi^2**	0
**Retained Factors**	6	**Number of params**	63
**N**	2171		

Phenotypic analysis

Supplementary Table 12: Associations of capped GCSE score with individual risk behaviours, based on complete case sample.

	Capped GCSE score		
**Risk behaviours**	**β**	**95% CI**	
Physical inactivity	-0.02	-0.10	0.06
TV viewing	-0.16	-0.25	-0.07
Car passenger risk	-0.25	-0.33	-0.17
Cycle helmet use	0.18	0.12	0.24
Scooter risk	-0.40	-0.49	-0.31
Illicit drug use/solvent use	-0.48	-0.64	-0.33
Cannabis use	-0.40	-0.53	-0.28
Regular tobacco use	-0.67	-0.76	-0.57
Hazardous alcohol consumption	-0.15	-0.22	-0.09
Self-harm	-0.22	-0.31	-0.12
Penetrative sex before the age of 16	-0.34	-0.43	-0.25
Unprotected sex	-0.36	-0.57	-0.16
Criminal and delinquent behaviour	-0.26	-0.31	-0.21

Supplementary Table 13: Associations of capped GCSE score with an index of the 13 original multiple risk behaviours, based on the complete case sample.

**Capped GCSE score**	**Model 1[a] N=1877**	**Model 2[b] N=1711**	**Model 3[c] N=1693**	**Model 4 [d] N=1583**
95% confidence intervals in brackets				
**MRB Index (13 behaviours)**	-0.07 [-0.09, -0.05]	-0.06 [-0.07, -0.04]	-0.06 [-0.07, -0.04]	-0.06[-0.07, -0.04]

[a] Model 1 is unadjusted for any covariates.
[b] Model 2 is adjusted for parental socioeconomic position, maternal education (ref:<O level) and sex (ref: male).
[c] Model 3 is adjusted for parental socioeconomic position, maternal education (ref:<O level), sex (ref: male) and housing tenure (ref: owned).
[d] Model 4 is adjusted for parental socioeconomic position, maternal education (ref:<O level), sex (ref: male), housing tenure (ref: owned) and cognitive ability.

Supplementary Table 14: Associations of capped GCSE score with an index of 11 risk behaviour that were highly correlated with the overall MRB Index scale.

**Capped GCSE score**	**Model 1[a] N=1910**	**Model 2[b] N=1739**	**Model 3[c] N=1721**	**Model 4[d] N=1608**
95% confidence intervals in brackets				
**MRB Index (11 behaviours)**	-0.07 [-0.07, -0.05]	-0.06 [-0.8, -0.04]	-0.05 [-0.08, -0.04]	-0.06[-0.08, -0.04]

[a] Model 1 is unadjusted for any covariates.
[b] Model 2 is adjusted for parental socioeconomic position, maternal education (ref:<O level) and sex (ref: male).
[c] Model 3 is adjusted for parental socioeconomic position, maternal education (ref:<O level), sex (ref: male) and housing tenure (ref: owned).
[d] Model 4 is adjusted for parental socioeconomic position, maternal education (ref:<O level), sex (ref: male), housing tenure (ref: owned) and cognitive ability.

Supplementary Table 15: Associations of capped GCSE score with first factor, based on complete case sample.

Capped GCSE score	Model 1[a] N=1877	Model 2[b] N=1711	Model 3[c] N=1693	Model 4[d] N=1583
95% confidence intervals in brackets				
**First Factor**	-0.16 [-0.20, -0.12]	-0.14 [-0.18, -0.10]	-0.14 [-0.17, -0.10]	-0.14[-0.17, -0.10]

[a] Model 1 is unadjusted for any covariates.

[b] Model 2 is adjusted for parental socioeconomic position, maternal education (ref:<O level) and sex (ref: male).

[c] Model 3 is adjusted for parental socioeconomic position, maternal education (ref:<O level), sex (ref: male) and housing tenure (ref: owned).

[d] Model 4 is adjusted for parental socioeconomic position, maternal education (ref:<O level), sex (ref: male), housing tenure (ref: owned) and cognitive ability.

GREML

Supplementary Table 16: Univariate model including for both MRB indexes, educational achievement measures, and individual risk behaviours.

Univariate estimates	n	h^2 [a]	SE	95% CI	
MRB Index (13 behaviours)	2171	0.18	0.15	-0.11	0.47
MRB Index (11 behaviours)	2171	0.28	0.15	-0.01	0.57
First Factor	2171	0.42	0.15	-0.13	0.71
Achieved 5 or more [b]	6709	0.34	0.05	0.24	0.44
Capped GCSE score [c]	6646	0.60	0.05	0.50	0.70
Physical inactivity	3556	0.08	0.09	-0.10	0.26
TV viewing	3584	0.08	0.09	-0.10	0.26
Car passenger risk	3547	0.18	0.09	0.00	0.36
Cycle helmet use	3227	0.16	0.10	-0.04	0.36
Scooter risk	3497	0.20	0.09	0.02	0.38
Illicit drug use/solvent use	3512	0.06	0.09	-0.12	0.24
Cannabis use	3578	0.17	0.09	-0.01	0.35
Regular tobacco use	3579	0.01	0.09	-0.17	0.19
Hazardous alcohol consumption	3399	0.14	0.10	-0.06	0.34
Self-harm	3582	0.21	0.09	0.03	0.39
Penetrative sex before the age of 16	3933	0.07	0.08	-0.09	0.23
Unprotected sex	3933	0.00	0.08	-0.16	0.16
Criminal and delinquent Behaviour	4017	0.07	0.08	-0.09	0.23

[a] h^2 shows the univariate heritability of each item
[b] Achieved 5 or more is a binary measure of educational achievement
[c] Capped GCSE score is a continuous measure of educational achievement

Supplementary Table 17: Bivariate estimates of educational achievement, different risk behaviours measures and first factor.

Bivariate estimates	n	r_g [a]	SE	95% CI	
Achieved 5 or more [b]: MRB Index (13 behaviours)	4440	-0.82	0.44	-1.68	0.04
Achieved 5 or more: MRB Index (11 behaviours)	4440	-0.51	0.25	-1.00	-0.02
Achieved 5 or more: First Factor	4440	-0.51	0.19	-0.88	-0.14
Capped GCSE score [c]: MRB Index (13 behaviours)	4409	-0.51	0.27	-1.04	0.02
Capped GCSE score: MRB Index (11 behaviours)	4409	-0.35	0.18	-0.70	0.003
Capped GCSE score: First Factor	4409	-0.43	0.14	-0.70	-0.16

[a] r_g shows the genetic correlation between two traits
[b] Achieved 5 or more is a binary measure of educational achievement
[c] Capped GCSE score is a continuous measure of educational achievement

MR

Supplementary Figure 3: Association between the young person's genetically-instrumented MRB Index (13 behaviours) and their educational achievement (capped GCSE score, standardised).

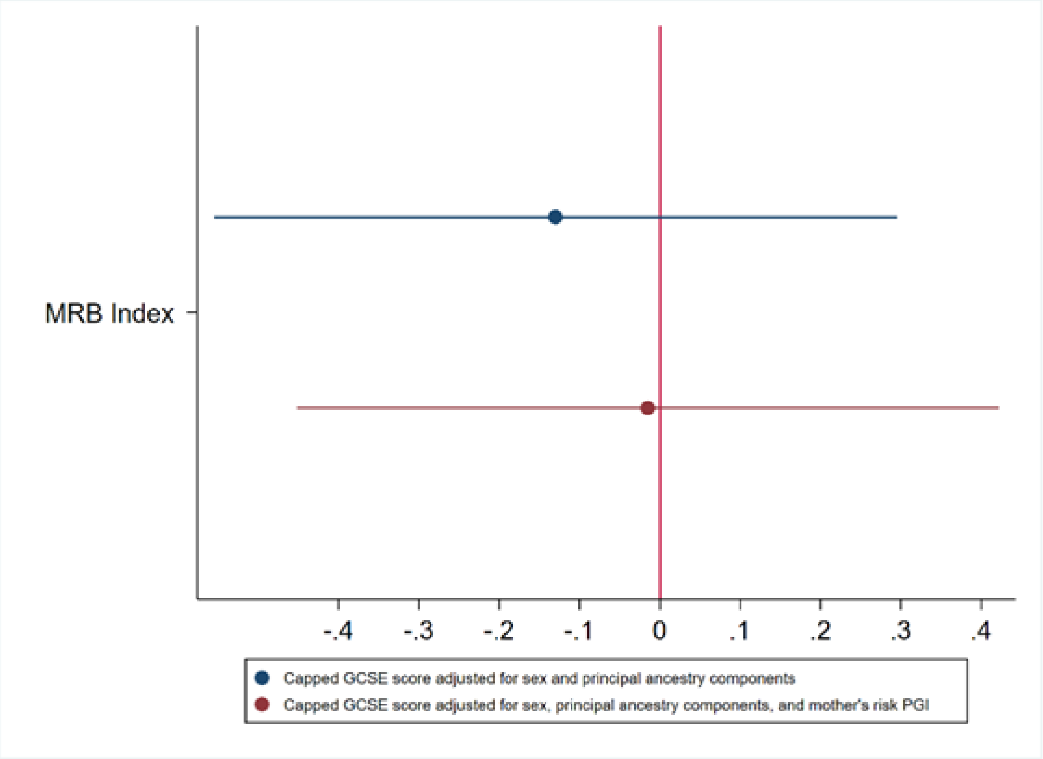

Supplementary Figure 4: Association between the young person's genetically-instrumented educational achievement (capped GCSE score, standardised), and their MRB Index (13 behaviours).

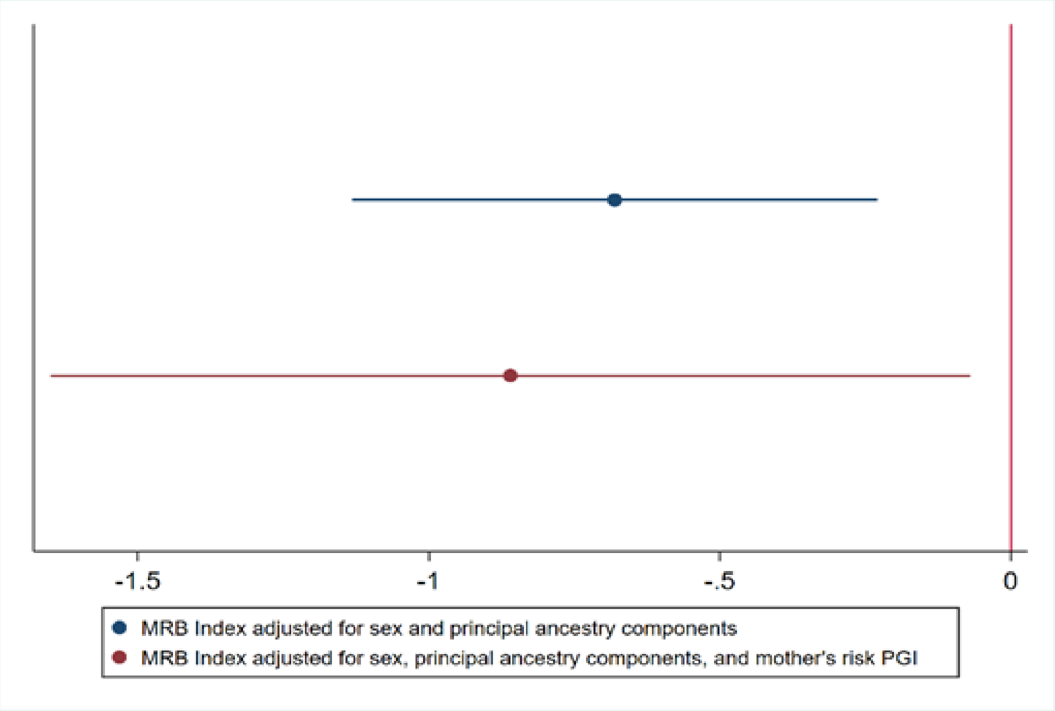

Supplementary Figure 5: Association between the young person's genetically-instrumented MRB Index (11 behaviours) and their educational achievement (capped GCSE points score, standardised).

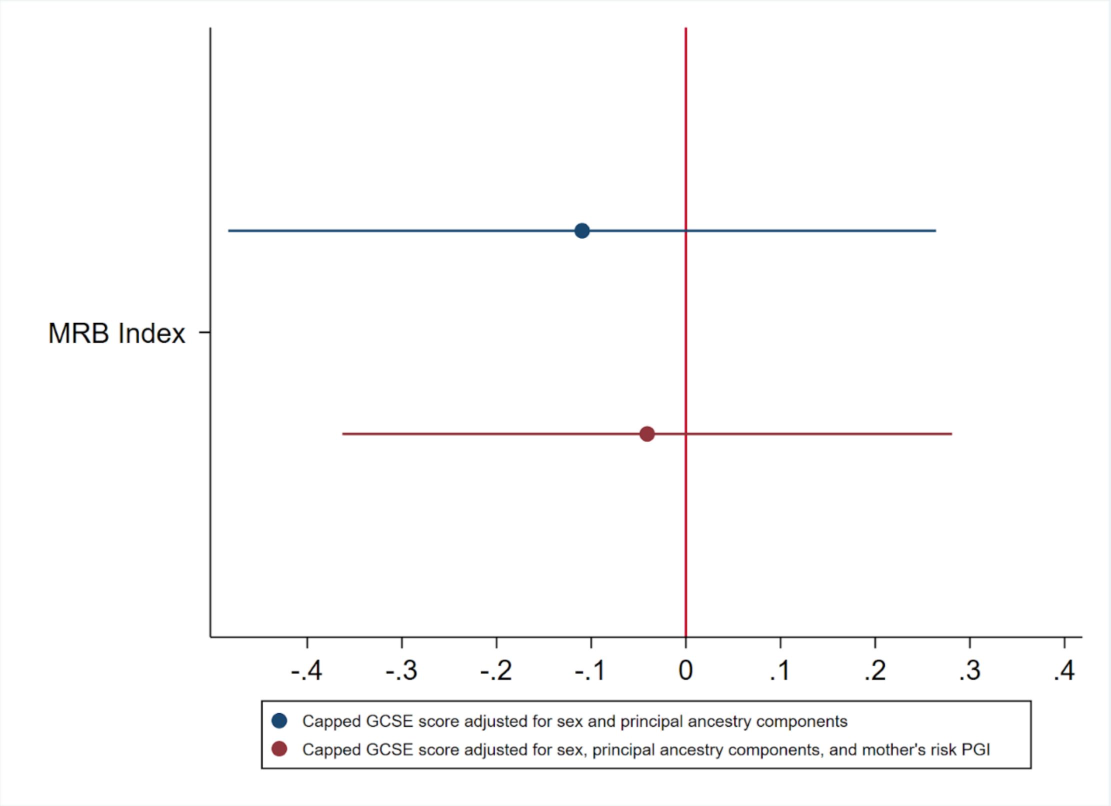

Supplementary Figure 6: Association between the young person's genetically-instrumented educational achievement (capped GCSE score, standardised), and their MRB Index (11 behaviours)

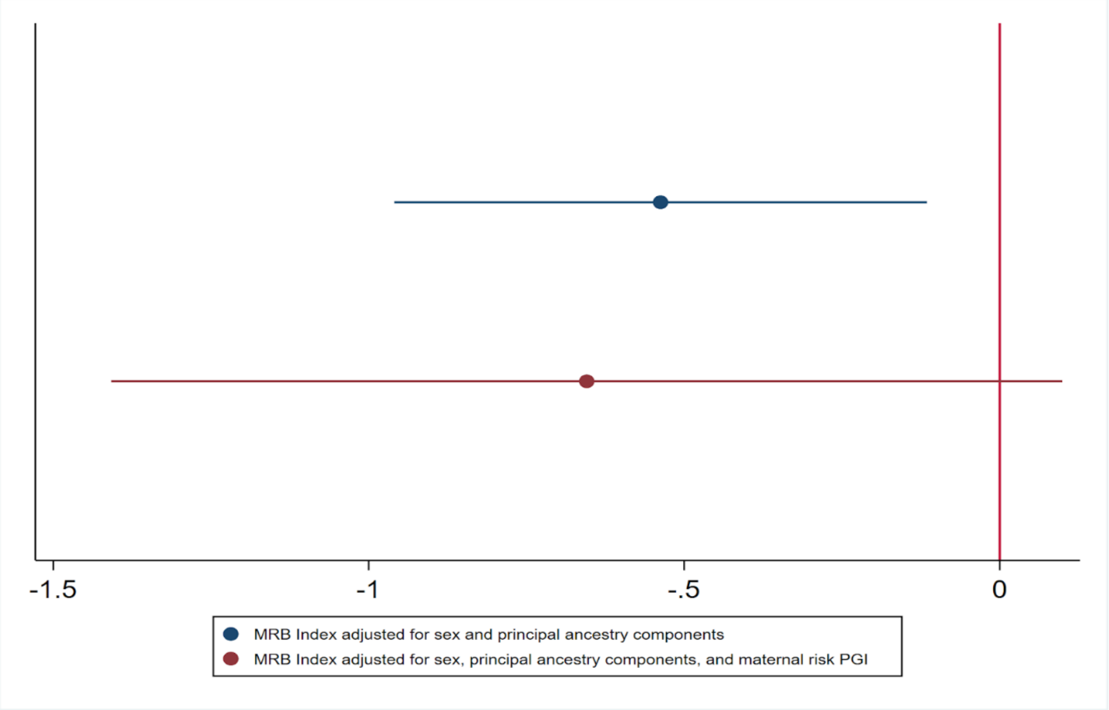

Supplementary Figure 7: Association between the First Factor of the young person's genetically-instrumented risk behaviours and their educational achievement (capped GCSE score, standardised).

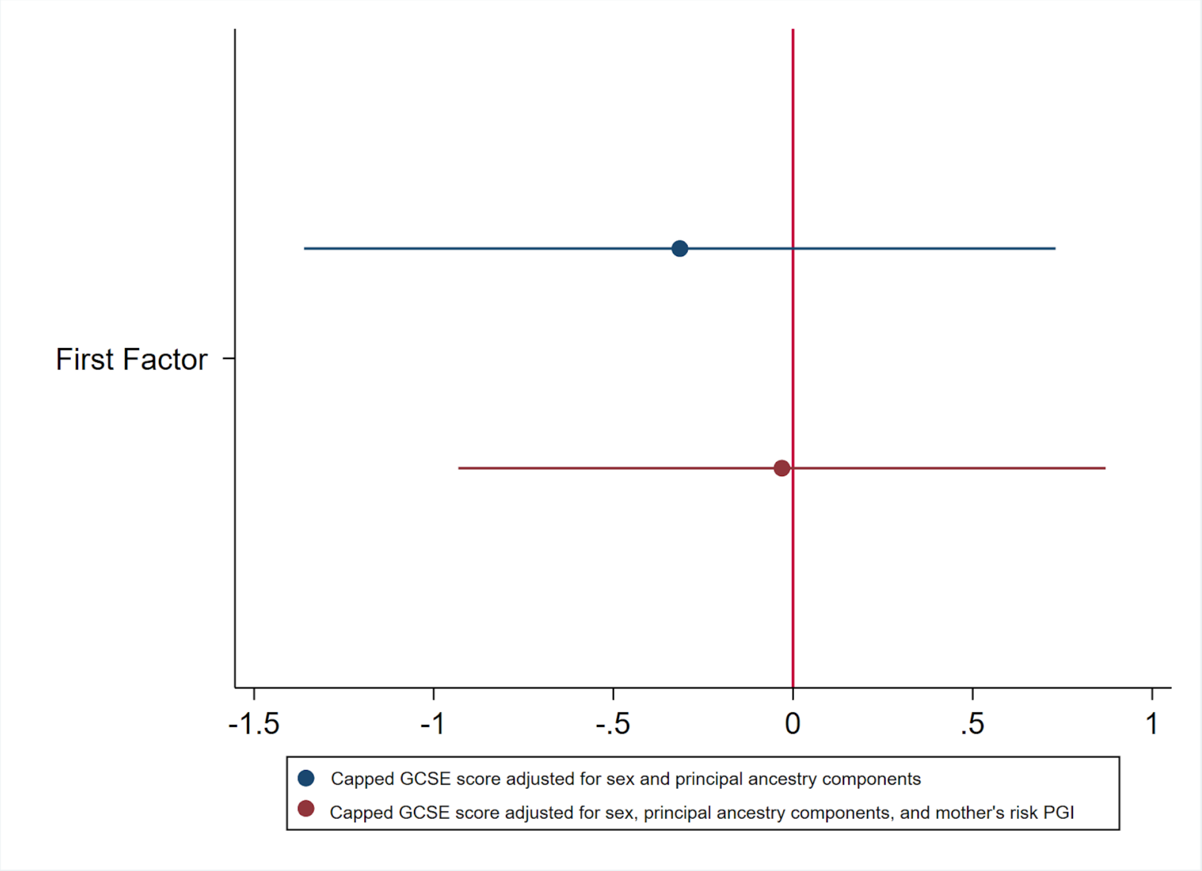

Supplementary Figure 8: Association between the young person's genetically-instrumented educational attainment (capped GCSE score, standardised), and the MRB Index first factor.

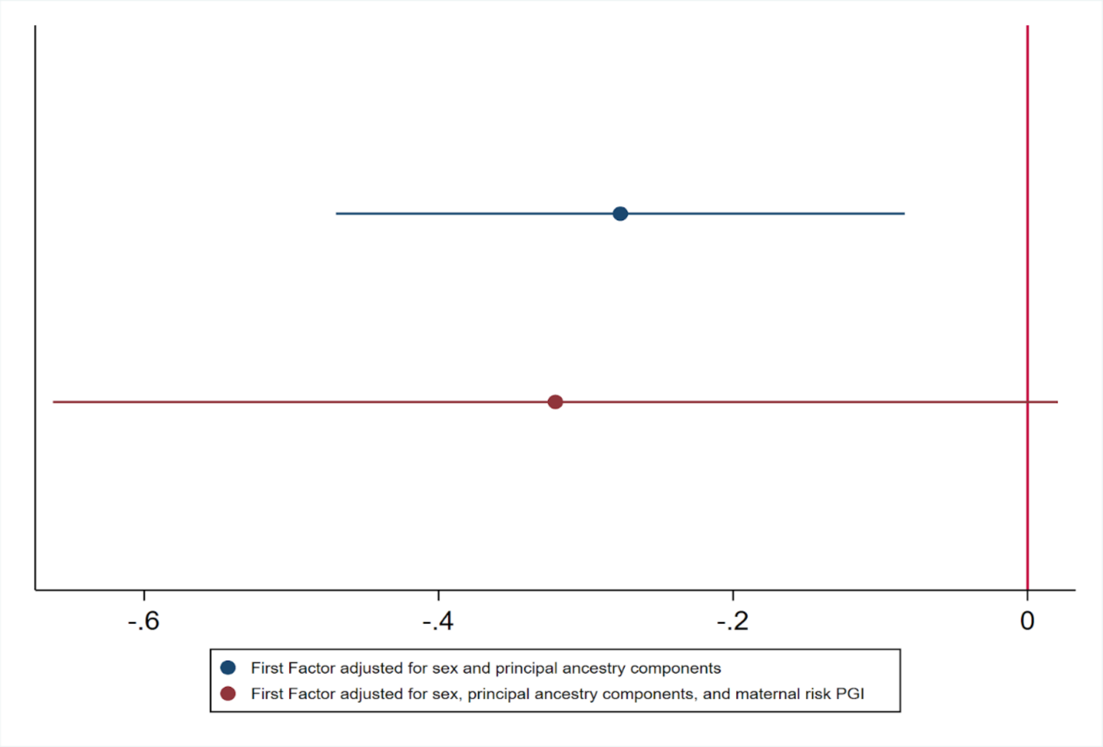

Supplement: Supplementary file 2 — Supplementary material [file 42003_2024_6091_MOESM2_ESM.pdf]
