# Supplementary material for: Genetic associations of risk behaviours and educational achievement
Source: Commun Biol. 2024 Apr 10;7:435. doi: 10.1038/s42003-024-06091-y (PMC11006670; doi:10.1038/s42003-024-06091-y)
Supplement: Supplementary file 3 — Reporting Summary [file 42003_2024_6091_MOESM3_ESM.pdf]

nature portfolio

Corresponding author(s): Michelle Arellano Spano

Last updated by author(s): Mar 19, 2024

Reporting Summary

Nature Portfolio wishes to improve the reproducibility of the work that we publish. This form provides structure for consistency and transparency in reporting. For further information on Nature Portfolio policies, see our Editorial Policies and the Editorial Policy Checklist.

Statistics

For all statistical analyses, confirm that the following items are present in the figure legend, table legend, main text, or Methods section.

n/a	Confirmed	
☐	☒	The exact sample size (*n*) for each experimental group/condition, given as a discrete number and unit of measurement
☐	☒	A statement on whether measurements were taken from distinct samples or whether the same sample was measured repeatedly
☐	☒	The statistical test(s) used AND whether they are one- or two-sided<br>*Only common tests should be described solely by name; describe more complex techniques in the Methods section.*
☐	☒	A description of all covariates tested
☐	☒	A description of any assumptions or corrections, such as tests of normality and adjustment for multiple comparisons
☐	☒	A full description of the statistical parameters including central tendency (e.g. means) or other basic estimates (e.g. regression coefficient) AND variation (e.g. standard deviation) or associated estimates of uncertainty (e.g. confidence intervals)
☐	☒	For null hypothesis testing, the test statistic (e.g. *F*, *t*, *r*) with confidence intervals, effect sizes, degrees of freedom and *P* value noted<br>*Give P values as exact values whenever suitable.*
☒	☐	For Bayesian analysis, information on the choice of priors and Markov chain Monte Carlo settings
☐	☒	For hierarchical and complex designs, identification of the appropriate level for tests and full reporting of outcomes
☐	☒	Estimates of effect sizes (e.g. Cohen's *d*, Pearson's *r*), indicating how they were calculated

Our web collection on statistics for biologists contains articles on many of the points above.

Software and code

Policy information about availability of computer code

Data collection	No software used for data collection.
Data analysis	Data analysis<br>Avon Longitudinal Study of Parents and Children (ALSPAC): STATA/17MP<br>GWAS reformatting to merge to ALSPAC data: R version 4.2.1<br>GWAS clumping: TwoSampleMR R package in R package<br>Polygenic index estimation: PLINK version 1.9<br><br>Code availability: All the code used to clean and analyse the data for this study is available: https://github.com/MichelleSpano/Risk-behaviours

For manuscripts utilizing custom algorithms or software that are central to the research but not yet described in published literature, software must be made available to editors and reviewers. We strongly encourage code deposition in a community repository (e.g. GitHub). See the Nature Portfolio guidelines for submitting code & software for further information.

Data

Policy information about availability of data

All manuscripts must include a data availability statement. This statement should provide the following information, where applicable:

- Accession codes, unique identifiers, or web links for publicly available datasets
- A description of any restrictions on data availability
- For clinical datasets or third party data, please ensure that the statement adheres to our policy

The informed consent obtained from ALSPAC participants does not allow the data to be made freely available through any third party maintained public repository. Data used for this submission can be made available on request to the ALSPAC Executive. The ALSPAC data management plan describes in detail the policy regarding data sharing, which is through a system of managed open access. Full instructions for applying for data access can be found here: http://www.bristol.ac.uk/alspac/researchers/access/.
The GWAS summary statistics for both risk behaviours and educational attainment used on the analyses are available through the Social Science Genetic Association Consortium (SSGAC). Available through the SSGAC website: https://www.thessgac.org/.

Research involving human participants, their data, or biological material

Policy information about studies with human participants or human data. See also policy information about sex, gender (identity/presentation), and sexual orientation and race, ethnicity and racism.

Reporting on sex and gender	Phenotypic analyses were controlled for sex. Of the original ALSPAC sample restricted by genetic data available (8,815), 51% were male and 49% female. The study results were not stratified by sex. Sex assigned by birth was used as provided in the ALSPAC dataset and collected in clinical measures.
Reporting on race, ethnicity, or other socially relevant groupings	We did not use race or ethnicity in our study. Socioeconomic measures were used as controls. We derived measures of: maternal education, housing tenure at time of child's birth and parental social class. We used self-reported information from ALSPAC questionnaires to derive these measures.
Population characteristics	Table 3 in the supplementary shows the characteristics and differences between our analytical sample, that had been restricted to those participants that had genetic information, all covariates and information on risk behaviour (N=1735) and the core ALSPAC sample (N=15,616). Based on the analytical sample, imputed the sample, and carried out the phenotypic analysis and MR analyses on the imputed sample (N=7,695).
Recruitment	No direct recruitment was carried out in this study.
Ethics oversight	Ethical approval for the study was obtained from the ALSPAC Ethics and Law Committee and the Local Research Ethics Committees. Consent for biological samples has been collected in accordance with the Human Tissue Act (2004) (for full information on ALSPAC ethical approval please see: http://www.bristol.ac.uk/alspac/researchers/research-ethics/).

Note that full information on the approval of the study protocol must also be provided in the manuscript.

Field-specific reporting

Please select the one below that is the best fit for your research. If you are not sure, read the appropriate sections before making your selection.

☒ Life sciences ☐ Behavioural & social sciences ☐ Ecological, evolutionary & environmental sciences

For a reference copy of the document with all sections, see nature.com/documents/nr-reporting-summary-flat.pdf

Life sciences study design

All studies must disclose on these points even when the disclosure is negative.

Sample size	The complete case sample was derived from the ALSPAC cohort, starting with 15,616 participants. This sample was then restricted to those who had genetic data available and were unrelated (N=8,815). Of the genetic sample, 2,366 participants had information on some risk behaviours. Finally, our analytical sample was composed of 1,735 who had complete genetic data, risk behaviours and full information on covariates (see supplementary Figure 1 for STROBE diagram). We carried out the phenotypic analyses in an imputed sample. In order to maintain the sample size, we imputed the missing values by chained equations in Stata. During the multiple imputation 50 datasets were created. Besides variables used in the analysis, we included marital status, mother's smoking status, maternal education, housing tenure and parental social class as auxiliary variables. We used logistic regression to impute the risk behaviours, linear and truncated regression for continuous variables and order logistic regression to impute categorical variables.
Data exclusions	Sample derivation and participant exclusion are illustrated in Figure 3 of the paper in the STROBE diagram. Of the 15,616 ALSPAC participants, we excluded 6,801 that did not have any genetic information. Then, further 6,449 were excluded as the participants did not have information on risk behaviours. Thus, the risk set was of 2,366, of which 631 were missing come covariates. Lastly, our complete case sample was of 1, 735. This sample was then imputed.<br>When creating the polygenic indexes, SNPs were excluded if they were palindromic at the harmonization step (i.e. the alleles on the foward

	strand are the same as the reverse stand) and were clumpped at a threshold of The clumping threshold was of r2=0.01 and LD=10,000.
Replication	We used three complimentary designs. We found evidence of an association between risk behaviours and educational attainment. This was further supported by GREML analysis, where we observed considerable genetic overlap between the traits. Lastly, the bidirectional Mendelian Randomization analysis explored the direction of the effect. Despite triangulation in our findings, replication in other international cohorts is necessary. No direct replication of each individual analysis was done.
Randomization	In the phenotypic analyses of the ALSPAC cohort we controlled for: sex, social class, maternal education, housing tenure at the time of the child's birth and cognitive ability. In the ALSPAC polygenic index analysis were controlled for the first 10 principal components to avoid population stratification bias. Mendelian Randomization exploits the random nature of the genetic variants used as instruments, thus it is often compared to a natural randomized controlled trial. This is similar to blinding at allocation.
Blinding	Blinding of the sample was not applied and not relevant as there were no interventions in this study.

Reporting for specific materials, systems and methods

We require information from authors about some types of materials, experimental systems and methods used in many studies. Here, indicate whether each material, system or method listed is relevant to your study. If you are not sure if a list item applies to your research, read the appropriate section before selecting a response.

Materials & experimental systems

n/a	Involved in the study
☒	☐ Antibodies
☒	☐ Eukaryotic cell lines
☒	☐ Palaeontology and archaeology
☒	☐ Animals and other organisms
☒	☐ Clinical data
☒	☐ Dual use research of concern
☒	☐ Plants

Methods

n/a	Involved in the study
☒	☐ ChIP-seq
☒	☐ Flow cytometry
☒	☐ MRI-based neuroimaging
