# Supplementary material for: Genetic associations of risk behaviours and educational achievement
Source: Commun Biol. 2024 Apr 10;7:435. doi: 10.1038/s42003-024-06091-y (PMC11006670; doi:10.1038/s42003-024-06091-y)
Supplement: Supplementary file 1 — Peer Review File [file 42003_2024_6091_MOESM1_ESM.pdf]

nature portfolio

Peer Review File

Genetic associations of risk behaviours and educational achievement

Open Access This file is licensed under a Creative Commons Attribution 4.0 International License, which permits use, sharing, adaptation, distribution and reproduction in any medium or format, as long as you give appropriate credit to the original author(s) and the source, provide a link to the Creative Commons license, and indicate if changes were made. In the cases where the authors are anonymous, such as is the case for the reports of anonymous peer reviewers, author attribution should be to 'Anonymous Referee' followed by a clear attribution to the source work. The images or other third party material in this file are included in the article's Creative Commons license, unless indicated otherwise in a credit line to the material. If material is not included in the article's Creative Commons license and your intended use is not permitted by statutory regulation or exceeds the permitted use, you will need to obtain permission directly from the copyright holder. To view a copy of this license, visit http://creativecommons.org/licenses/by/4.0/.

REVIEWERS' COMMENTS:

Reviewer #1 (Remarks to the Author):

Thank you for addressing the reviewer's comments so thoroughly and carefully. The paper is much stronger now; the thorough robustness checks should be commended.

Reviewer #3 (Remarks to the Author):

The authors have now addressed my previous comments on this manuscript. I still somewhat feel that the impact of the paper is lost in its lack of detail on interventions or actions from the findings (which other reviewers highlighted), however, I do agree that the rewritten introduction hints better at real-world implications than before. Subsequently, I do not have further detailed comments for the authors.
